# Folie et Société: eroding the body–mind relationship via dysfunctional paternalistic systems

**DOI:** 10.3389/fpsyg.2024.1324303

**Published:** 2024-02-05

**Authors:** Matt Hudson, Nazish Idrees Chaudhary, Curtis Nordstrom

**Affiliations:** ^1^Mind Help Limited, Durham, United Kingdom; ^2^Lahore School of Behavioural Sciences, The University of Lahore, Lahore, Pakistan; ^3^Psychiatrie Baselland, Liestal, Basel-Landschaft, Switzerland

**Keywords:** Folie et Société, split second unlearning, body-mind relationship, emotional memory images, dysfunctional paternalistic systems, family systems and functioning, body dysmorphic disorder, anorexia nervosa

## Abstract

This theoretical perspective examines the proposition of shared complex trauma between a parent and child, arising from blurred relational boundaries and societal oppression, leading to inequality both at home and within the larger paternalistic system of society. Specifically, the focus is on living within a paternalistic, authoritarian system where rules are unjust, demanding obedience and compliance without questioning the behaviors of the authority. Individuals growing up in these circumstances are subject to adverse and emotionally overwhelming experiences, which lead to the creation of emotional memory images (EMIs). The delusion in which the child is caught up becomes a reality for the child as time passes. This phenomenon is recognized in psychiatry as “Folie à deux” (the madness of two or more) at the micro level, and “Folie et Société” (the madness of society) on the macro level. Complex trauma, derived from a child’s exposure to multiple adverse events, can erode the mind–body relationship, impacting both mental and physical health. These traumatic experiences in early childhood can manifest as body-focused disorders in adolescents, prevailing throughout adulthood if left unattended. This article provides a theoretical perspective on dealing with the dissociation and chronic stress related to oppressive and authoritarian family systems. The broader implications of this article include highlighting the psychophysiological underpinnings of complex trauma, the relationship of a highly oppressive paternalistic authoritarian system imposed on children and adolescents, and the role of Split-Second Unlearning as a therapeutic intervention to clear EMIs and improve overall health outcomes.

## Introduction

Research spanning decades has established a link as strong as the connection between smoking and lung cancer, demonstrating that child maltreatment significantly affects social, behavioral, mental, and physical health throughout a person’s lifetime (Bentall et al., 2014). Psychological or emotional abuse, often a prevalent yet overlooked form of child maltreatment, has only recently gained more attention (Dube et al., 2023). There are firm links between childhood trauma and the sequelae in adult health (Afifi et al., 2016; Herzog and Schmahl, 2018). Adverse childhood experiences (ACEs) (Centers for Disease Control and Prevention, 2021) not only heighten the likelihood of mental health issues but also leave a lasting impact on the body, increasing the risk of physical illnesses and even death (Hughes et al., 2017). Specifically, individuals who have experienced childhood trauma are more prone to developing somatic ailments like musculoskeletal pain, ear, nose, and throat issues, gastrointestinal problems (Kirmayer et al., 2004), fatigue, and dizziness (Ho et al., 2021). These issues often evolve into chronic conditions like fibromyalgia (Pinto et al., 2023), chronic fatigue (Afari et al., 2014), irritable bowel syndrome (Sansone and Sansone, 2015) and psychosis (Croft et al., 2019; Loewy et al., 2019; Karcher et al., 2020). A plethora of growing evidence suggests that experiences of trauma in childhood may contribute to the development of psychosis (Croft et al., 2019; Loewy et al., 2019; Peach et al., 2019; Karcher et al., 2020), which can be defined by abnormalities within the following categories: delusions, hallucinations, disorganized thoughts, disorganized behavior and negative symptoms (Calabrese and Al Khalili, 2023). Indeed, children of parents with major psychoses often exhibit similar cognitive dysfunctions, and childhood maltreatment further elevates the risk of adult psychoses through unclear mechanisms (Berthelot et al., 2015).

This chronic early trauma or complex trauma results from multiple, interpersonal, adverse events during childhood, undermining a typical caregiving relationship (Wamser-Nanney and Vandenberg, 2013; Kliethermes et al., 2014). Individuals raised in a dysfunctional family environment marked by shared delusions, or Folie à deux, may become predisposed to a wide array of emotional, psychological, neurological, behavioral, and physiological disorders (Hudson and Johnson, 2023a). Folie à deux is a delusional disorder that falls under the mental illness category. It has been repositioned from its previous classification in the Diagnostic and Statistical Manual of Mental Disorders, Fifth Edition (DSM-5), to now be a part of the Other Specified Schizophrenia Spectrum and Other Psychotic Disorders category (Bjelan et al., 2022). The DSM-5 lists ten personality disorders, all of which describe afflicted individuals as having a “*rigid and unhealthy pattern of thinking*.” (Chapman et al., 2023) To date, DSM-5 defined disorders have no scientific etiological basis for pathology, diagnosis, or pharmacological treatment, indeed, no biological basis for these conditions is thought to exist (Scull, 2021). This article aims to shed new light on the influence of dysfunctional familial interactions on mental health conditions in the hope of reducing the shadow that is cast upon the lives of individuals who suffer from this often debilitating disorder. From the initial seed of dysfunction and shared delusion various disorders may flourish, including but not limited to anxiety disorders, depression, post-traumatic stress disorder (PTSD), personality disorders or even certain somatic disease states. For instance, those with Borderline Personality Disorder (BPD) display a heightened sensitivity to social cues, avoiding them more frequently and being more conscious of this behavior compared to the general population. If a caregiver fails to perform the vital role of fostering a sense of continuous existence, the infant may struggle to develop a genuine sense of self (Winnicott, 1965). As a result, the infant may construct a false self, shaped by external pressures. This disruption in the sense of continuous existence leads to the formation of a defensive mechanism aimed at managing the profound distress of disintegration and psychological annihilation (Ogden, 2014).

## Theories underpinning this article

Theories that are central to this article surrounding complex trauma:

Structural Dissociation (Steele et al., 2005), which explains the division within the self that results from trauma. Attachment Theory (Bowlby, 1979), which describes how early bonds with caregivers can influence an individual’s reaction to trauma.

Traumatic Bonding theory (Dutton and Painter, 1993), also plays a role, detailing the strong emotional connections that can form in abusive relationships.

Split-Second Unlearning (SSU) (Hudson and Johnson, 2021), informs us that psychophysiological stress arises from significant emotional events, leading to chronic conditions like anxiety and fibromyalgia. These events generate emotional memory images (EMIs) that are formed and reinforced within moments—split-second learning. Daily triggers can reactivate these EMIs, causing repeated stress responses and chronic psychophysiological dis-ease. Clients learn to dissociate EMIs from stress responses by becoming observant of their nonverbal responses and “unlearning” or emotionally detaching from the memory.

## Folie à Deux delusional beginnings

Folie à deux is a concept coined in 1877 by two French physicians, Lasègue and Falret. It is also called Lasegue-Falret syndrome [Lasegue and Falret, 1877, as cited in Saragih et al. (2019)]. The presence of a shared psychotic disorder between two or more people is commonly observed, such as in cases of schizophrenia and delusional disorder (Kovacevic et al., 2022). In these instances, a person (inducer) induces a delusion to others in society or a household. This condition includes the transfer of delusional ideas from one person to another (Srivastava and Borkar, 2010) when the primary affected person transfers their psychosis to those with whom they share a close relationship (Tsarkov, 2020; Kawasaki, 2022).

The role of a dominant, authoritative, and controlling oppressor is assumed by the delusional caregiver. This abuse of the parental relationship creates emotional overwhelm within the infant or child, leading to the creation of emotional memory images (EMIs) (Hudson and Johnson, 2022). Whenever a similar situation occurs, the EMIs activate the original response that the amygdala selected in order to survive. The presence of the EMI can drive the concept of self from the body, creating a dissociative, trance-like state (Saragih et al., 2019). This may appear to be a flight response, yet it is more akin to a freeze mechanism, as the self remains hypervigilant waiting for the proposed threat to clear (see Kozlowska et al., 2015 for more on fear defence cascade).

According to the World Health Organization (2022), three-quarters of children have faced maltreatment from their parents or caregivers. This can disrupt an individual’s sense of self, both mentally and physically, hindering the ability to connect with others, and may even affect consciousness so profoundly that one might psychologically detach from the body for survival. This separation from self can lead to body dysmorphic disorder (BDD), whereby individuals become preoccupied with perceived defects in their physical appearance (American Psychiatric Association, 2013). Specifically, theories suggest that emotional abuse might lead to deep-seated self-criticism in BDD, whereas physical or sexual abuse could be linked to shame, centered on one’s physical appearance (Veale and Neziroglu, 2010). Such prolonged inner processes require an intervention to prevent the development of dissociative pathologies and disorders at an early stage among children from these environments (Sar, 2022).

## Dysregulation, or a natural, rational response to a nonconscious threat?

The fear defence cascade, first identified in animal studies, shows specific threat responses; flight, fight (hyperarousal), freeze, tonic immobility, and quiescent immobility (hyporarousal). These survival states are highly adaptive and adjust depending on the proximity of the animal and the predator, thus stress is naturally regulated (see Kozlowska et al., 2015 for an in-depth review). The freeze response is of particular interest as it activates when animals sense a predatory threat, or in lab settings when they encounter environments or specific signals linked to past negative experiences (Kalin and Shelton, 1989; Carrive, 2006). Attentive immobility affords the animal the ability to scan the immediate area for the presence of a predator or prey (the visual cortex is wired for movement in mammalians), while remaining perfectly still or frozen (Bracha, 2004).

Unlike animals, the perceived threat from an EMI within the mind’s eye presents an illusion to the brain, activating an appropriate heuristic stress response to the original threatening time and space (Hudson and Johnson, 2022). This activation of the mind and body is commonly reported as dysregulated stress (Roberts and Karatsoreos, 2021). Although the induced individual’s reaction to the EMI is genuine, when witnessed by an external observer the response may appear dysregulated and delusional. An individual’s behavior at this moment in time would clearly demonstrate detachment from the self and a breakdown of mental functioning.

## The mind’s eye sees the known unknown

**Figure 1:**
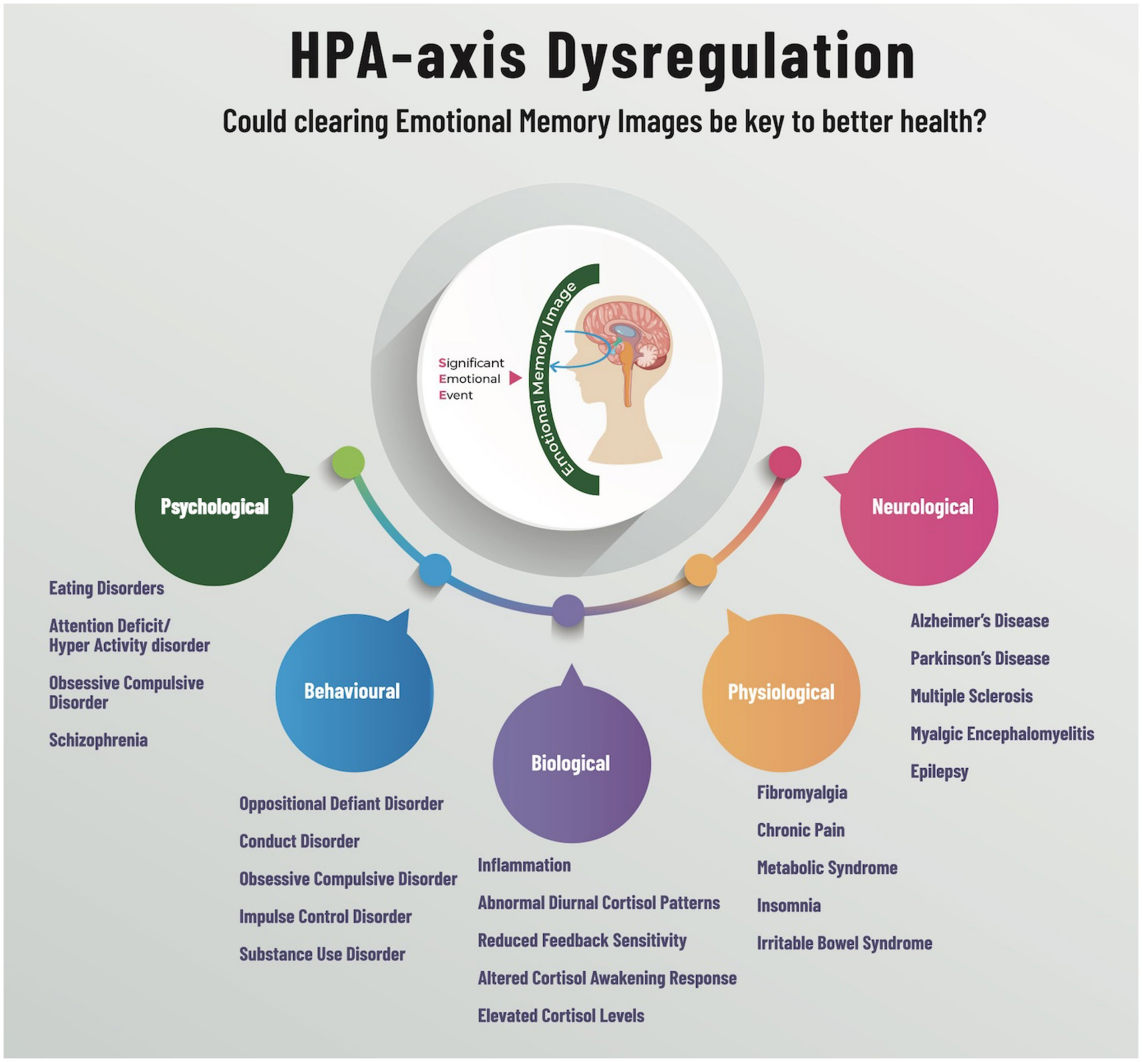
The impact of split-second unlearning.

In animal studies, the fear defence cascade operates sequentially depending on the proximity of the prey or predator. The animal kingdom needs to see, smell, or hear the threat in order for the automatic responses to engage and subsequently disengage after the threat has passed. However, what if the predator remains? Flight or fight are then associated with an active state of hyperarousal, while the passive hyporarousal state is associated with freeze. The freeze response is also a state of hypervigilance. The authors assert that the distinction between animal and human responses to fear is that humans have created an EMI. Therefore, EMIs are the upstream stressor that initiates context or content-specific psychophysiological responses (see Figure 1). For instance, when a parent or caregiver shouts at a child who is interacting with a spider, saying, “Do not touch the spider!” it creates an emotionally charged memory imprint (EMI) of fear in the child. Before being yelled at, the child was not fearful of the spider. This newly formed EMI leads to a fear-avoidance response toward spiders, which serves to enhance safety and increase the chances of survival in subsequent interactions.

Freud proposed traumatic memory is actively running in the background as a defence (Freud, 1958). From an evolutionary point of view, the brain defends against the EMI. Although the subconscious mind is totally alert and aware of the threat posed by the EMI, the individual is not. Therefore, the body remains still or numb and the eyes either fixate on or avert from the EMI. Eye fixation or aversion are both indicators of a social anxiety disorder (SAD) and fear (Chen et al., 2020). The induced now views life through an EMI that is situated between reality and the perception of reality. All social interactions, therefore, have the propensity for EMI activation, leaving the induced open to both real and imagined threats as they encounter different facial (visual) and tonal (auditory) cues (Noordewier et al., 2020). The authors propose that the EMI filter creates and supports the child’s survival in the short term (for review see Machremi et al., 2022). However, one of the medium- to long-term effects of perceiving life through a delusion is body dysmorphic disorder (BDD). Notably, this disorder is associated with a 48% lifetime hospitalization rate and a concerning heightened risk of early mortality. Additionally, 24–28% of individuals with this condition attempt suicide at some stage in their lives (Feusner et al., 2010).

## Pathophysiological and behavioral development of body-focused disorders

The constant presence of an EMI for a subject within a dysfunctional family environment can be evidenced by the underutilization of several structural connections within the visual cortex. Body Dysmorphic Disorder (BDD) subjects have shown unusually low information transfer between the primary and secondary visual cortex areas, as well as within the advanced temporal lobe visual processing systems (Leow et al., 2013). Could it be that the psyche is attempting to minimize the visual component of the EMI in order to make life more bearable? The ventral visual stream (VVS) and the amygdala also share a relationship with the prevalence of BDD (Bohon et al., 2012). This is consistent with fMRI data which demonstrated hypoactivity in dorsal visual and parietal networks within BDD subjects observing photographs or faces. The findings of this study, which included anorexia nervosa (AN) subjects, may hold significance for comprehending the differing and common pathophysiological mechanisms that underlie perceptual distortions of appearance (Moody et al., 2021).

The authors suggest that the presence of an EMI prohibits the individual from seeing their true reflection in the mirror. Although outside of the individual’s awareness, an EMI is able to provoke feelings of self-loathing and shame as the contents of the EMI will contain the source of their delusion. Therefore, hyperarousal (fight or flight) is present in AN subjects, where the individuals have learned only to trust their own internal voice and imagery, over the distrust of their hypocritical, delusional caregivers. This may explain how AN patients often fail to see that they are painfully thin and fail to respond to their caregivers’ requests for them to eat. Hyporarousal is more prevalent in BDD subjects as they are more akin to communicated psychosis, where although the subjects may be separated from the inducers they are nevertheless still held under their influence (Al Saif and Al Khalili, 2023).

## First-time learning and dysfunctional parenting

The SSU theory suggests that EMIs act as extracorporeal bookmarks stored inside the mind’s eye, yet outside of the physical body. This nonconscious threat is theorized to cause the hypothalamic pituitary adrenal- axis (HPA-axis) to activate the body’s alarm system. When an EMI is initially formed, individuals experience situations that are intensely emotional and beyond their control, which would trigger a freeze response initiated by the HPA-axis. Activities that involve elements of both uncontrollability and social evaluation lead to significant alterations in cortisol and adrenocorticotropin hormone levels, as well as extended periods of recovery (Dickerson and Kemeny, 2004). EMIs have been defined as: “*Trauma induced, non-conscious, contiguously formed, multimodal, mental imagery, which trigger(s) an amnesic, anachronistic, stress response within a split-second*.” (Hudson and Johnson, 2022) The SSU model suggests that the continual activation of this bodily stress response can, over time, lead to various conditions that adversely impact the mind–body relationship, mental health function, and the development of body-focused disorders in adolescence, as well as a myriad of psychophysiological conditions (Hudson and Johnson, 2023b).

Treatment with SSU identifies a non-verbal marker of a subconscious stress response resembling a “freeze” state. In this method, clients actively participate as observers of their own reactions, noting these cues as they occur. By deconstructing the automatic responses, often linked to conditioned reflexes, the client is encouraged to separate their emotional and mental involvement from their physiological stress response, effectively leading to rapid desensitization or “split-second unlearning” (Hudson and Johnson, 2021).

## Folie et Société; a delusional paternal system

From an evolutionary standpoint, children depend on parents or caregivers for survival, exhibiting behaviors that foster alliances with those who offer protection and sustenance. Noncompliance with parental or caregiver directives could significantly jeopardize a child’s health and well-being, potentially leading to adverse or even fatal consequences. Therefore, the induced may obey rules that make no logical sense to an outside observer (Hudson and Johnson, 2023b).

On a larger scale, the SARS-Cov2 pandemic saw the widespread utilization of fear and psychological nudges (Almqvist and Andersson, 2021) by governmental “caregivers” to secure public compliance. Governmental authoritarian actions during crises may induce widespread societal distress, extending well beyond family dynamics. In these instances, government and other public agencies may assume a paternalistic, parental role as inducer for broad swathes of the public at large. The targeted utilization of captive media outlets, as well as federal and state officials, could then effectively initiate and maintain fear among the populace to support the aforementioned power imbalance, as well as elements of overall societal dysfunction (Wong et al., 2020; World Health Organization, 2020). Thus, the micro (Folie a Deux) delusion may become macro (Folie et Société). Indeed, the U.K. Minister for Health was quoted as saying that he wanted to “frighten the pants off everyone” during the SARS-Cov2 pandemic, initiating a joint media and government campaign intended to terrify the British public into compliance with hypocritical, dystopian rules (Diver, 2022; Forrest, 2023). The U.K. Prime Minister was also found guilty of misleading the House during the pandemic, where the decisions of many politicians were affected (Jones, 2022; Lilly, 2022).

Lockdowns and quarantines create social isolation and psychological distress for the vast majority who experience them (Brooks et al., 2020; Catty, 2021). In many countries, Folie et Société has continued to affect public opinion and behavior well after the declared end of the pandemic. Indeed, members of the public who disagree with the narrative of the paternalistic system of controls may be viewed by some of the induced populace as suffering from antisocial personality disorder (Black, 2022). However, does it really mean that they are crazy?

## Limitations

One of the major limitations of the paper is the unavailability of pilot testing data and results that may demonstrate the usefulness of SSU model in the management of certain psychophysiological conditions. Another limitation may be seen in the development of further guidelines to utilize the SSU model in order to deal with health conditions worsened by chronic traumatic symptoms.

As a theoretical perspective, the paper cannot yet provide these data. Further investigation and study are vital for the identification of EMIs, as well as the determination of efficacy with SSU to treat traumatic sequelae.

## Broader implications

- The SSU model’s application to biological and somatic systems could aid professionals in developing effective treatments for childhood trauma sequelae and complex PTSD.
- Mitigating the effects of traumatic symptoms could lessen psychopathology risks in adults with histories of family dysfunction and abuse.
- Effective treatment of early childhood trauma could dramatically diminish the demonstrated increased rates of mortality and morbidity from physical health issues.
- Comparing various factors impacting physical health and inflammatory diseases could highlight the need for trauma-informed clinical approaches to managing the litany of physical health conditions pursuant to trauma sequelae.

## Future research directions

The new, ICD-10 identifier for PTSD now identifies the diagnosis specifically as an emotional or fear-related disorder, whereas the previous diagnostic criteria of trauma with a variety of symptoms and almost six categories are now regarded as complex trauma. Childhood trauma and its health impacts on adulthood are pervasive across geographical and socioeconomic settings. Therefore, it is recommended that studies to validate the applicability of this intervention should be established.

## Conclusion

This article discusses the complex trauma arising from living under paternalistic, authoritarian systems both in family settings and broader society — environments which may be characterized by oppressive rules and blurred boundaries. These settings may foster “Folie à Deux” and “Folie et Société,” psychiatric phenomena where delusions are shared between two people or among a society, respectively. That which is classed as a mental health disorder may be considered a natural response to a persistent EMI, or an individual pushing back at the homogenized reality of paternalistic 21st-century societal intrusions. For the former, identifying the presence of an EMI is paramount, while for the latter, validating the incongruence of the paternalistic system is a starting point for improvement and change.

## Data availability statement

The original contributions presented in the study are included in the article/supplementary material, further inquiries can be directed to the corresponding author.

## Author contributions

MH: Conceptualization, Writing – original draft. NC: Writing – original draft. CN: Writing – review & editing.
